# Transcriptional Profile Changes after Noise-Induced Tinnitus in Rats

**DOI:** 10.3390/brainsci13040573

**Published:** 2023-03-29

**Authors:** Peng Liu, Xinmiao Xue, Chi Zhang, Hanwen Zhou, Zhiwei Ding, Li Wang, Yuke Jiang, Weidong Shen, Shiming Yang, Fangyuan Wang

**Affiliations:** 1 Medical School of Chinese People’s Liberation Army (PLA), Beijing 100853, China; 2 Department of Otolaryngology, Head and Neck Surgery, Institute of Otolaryngology, Chinese PLA General Hospital, Beijing 100853, China; 3 National Clinical Research Center for Otolaryngologic Diseases, State Key Lab of Hearing Science, Beijing Key Lab of Hearing Impairment Prevention and Treatment, Ministry of Education, Beijing 100853, China

**Keywords:** tinnitus, transcriptional profile, RNA-seq, genes

## Abstract

Tinnitus is an unpleasant symptom characterized by detective hearing without the actual sound input. Despite numerous studies elucidating a variety of pathomechanisms inducing tinnitus, the pathophysiology of tinnitus is not fully understood. The genes that are closely associated with this subtype of the auditory hallucination that could be utilized as potential treatment targets are still unknown. In this study, we explored the transcriptional profile changes of the auditory cortex after noise-induced tinnitus in rats using high throughput sequencing and verification of the detected genes using quantitative PCR (qPCR). Tinnitus models were established by analyzing startle behaviors through gap pre-pulse inhibition (PPI) of the acoustic startle. Two hundred and fifty-nine differential genes were identified, of which 162 genes were up-regulated and 97 genes were down-regulated. Analysis of the pathway enrichment indicated that the tinnitus group exhibited increased gene expression related to neurodegenerative disorders such as Huntington’s disease and Amyotrophic lateral sclerosis. Based on the identified genes, networks of protein–protein interaction were established and five hub genes were identified through degree rank, including *Fos, Nr4a1, Nr4a3, Egr2,* and *Egr3*. Therein, the *Fos* gene ranked first with the highest degree after noise exposure, and may be a potential target for the modulation of noise-induced tinnitus.

## 1. Introduction

Tinnitus refers to a phantom sensation that occurs when sound is perceived by hearing-loss patients and healthy people in the absence of actual external sounds [1]. Characteristics of the phonism vary among diverse people, and include ringing, buzzing, hissing, and sizzling, as well as complex phenotypes such as musical and even vocal hearing [2]. Previous work indicates that about 50 million US adults experience various degrees of tinnitus [3]. One-third of these patients seek medical assistance to alleviate the symptoms [3]. Meanwhile, the quality of life of an estimated 2 million residents in the USA is severely worsened by this intractable disease, which is comorbid with psychological illnesses such as depression and anxiety [4]. Tinnitus can be induced by multiple causes including loud noise exposure, ototoxic drug application, neuropsychiatric disorders, microcirculatory disturbance, and other unknown causes [5,6,7]. Although the etiology of tinnitus is intricate, major risk factors associated with this disorder have been proven to be hearing impairment and aging [8,9]. However, not everyone exposed to these risk factors acquires tinnitus, suggesting a genetic contribution to its generation and development [10]. Although research involving transcriptome analysis of tinnitus identified several genes related to the symptom [11,12,13], genetic markers of noise-induced tinnitus are still insufficient. Elucidating the potential genes underlying tinnitus susceptibility is imperative for the prevention and treatment of tinnitus patients.

Previous studies using peripheral hair cell deafferentation reported that animals with tinnitus exhibited higher ribbon loss than their counterparts without tinnitus [14,15]. Although the phantom sound is heard in the ear, tinnitus is not directly correlated with the impairment of the cochlea since the transection of the auditory nerve does not prevent the persistence of sound percept [16]. Researchers now are more inclined to attribute the generation of tinnitus to the imbalance of structures in the central nervous system. The auditory cortex, an essential center for sound management and perception, is considered as the primary region for tinnitus induction [17,18,19,20]. However, literature and evidence about genetic changes in the auditory cortex after tinnitus, especially that induced by loud noise, are scarce.

In the current work, we applied the RNA-seq technique to identify the different gene expressions associated with noise-induced tinnitus and to clarify essential signaling pathways involved in it. Primary genes were acquired in the central nervous system which may provide a promising avenue for exploring effective therapy for tinnitus patients.

## 2. Materials and Methods

### 2.1. Animals

Sprague-Dawley rats (2 months old) weighing 290–330 g in standard cages, with appropriate temperature (22 °C) and humidity (50–60%), were fed with freely acquired water and food in a 12-day/night cycle. The composition and source of the standard diet used in the present investigation were in accordance with implementing standard GB 14924.3-2010 (Laboratory animals—Nutrients for formula feeds). Given that startle magnitudes fluctuate throughout the estrous cycle in female animals [21,22], only male rats were selected as experimental subjects to rule out the effects of sex difference on the detection of tinnitus. The procedure executed in the research was in compliance with requirements of the Care and Use of Laboratory Animals in Chinese PLA General Hospital. The protocol applied in this study was approved by the Chinese PLA General Hospital Animal Ethical Committee.

### 2.2. Auditory Brainstem Responses (ABRs)

ABR testing was investigated to screen out animals with the intact hearing ability and to verify the noise exposure effect [23,24]. Briefly, sodium pentobarbital (40 mg/kg) was administrated to animals intraperitoneally (i.p.) to prevent narcosis. The animals were put into a chamber decorated with soundproof materials. A TDT loudspeaker (Tucker Davis Technologies, Miami, FL, USA) with a tube extended into the auditory canal was linked with a TDT RZ6 instrument to deliver the tone and click stimuli with a duration of 5 ms (0.5 ms rise/fall). During ABR testing, three needle electrodes (Rochester Electro-Medical, Lutz, FL, USA) comprising the active, the reference, and the ground needles were applied. The recording needle was inserted into the skin on the vertex, while the reference and ground needles were placed on the tested and contralateral mastoid, respectively. Tone (4, 8, 16, 32 kHz) and click stimuli were started at 90 dB sound pressure level (SPL) with a decreasing interval of 10 dB SPL. ABRs were amplified and filtered with a passband of 100 Hz to 3 kHz and average of 512 times. The threshold of ABR was considered as the lowest dB SPL of every frequency with repeatable wave III.

### 2.3. Gap Detection

To verify the induction of tinnitus among rats, the behavioral paradigm of gap-induced pre-pulse inhibition (PPI) of acoustic startle response was applied before and 1 week after noise exposure as described in a previous study [25,26]. In brief, rats were placed in a cage connected to a piezoelectric transducer (Xeye, Beijing, China) which could sense the change in pressure caused by an acoustic startle and instantly convert it into a voltage value. Sixty dB SPL narrow band background noise (6, 12, 16 kHz) inserted with a startle stimulus (115 dB SPL, 50 ms) was set as the “no Gap” pattern. The gap pattern was formed by delivering a silent gap (50 ms) ahead of the startle stimulus with an interval of 100 ms. The auditory stimuli were generated by a speaker above the platform (20 cm) which was controlled by custom Xeye startle software. All the tests were carried out in a random order composed of 10 paired “Gap” and “no Gap” trials. The gap detection ability was evaluated by Gap-PPI (%) calculated with the formula “1-Gap acoustic startle response amplitude/no Gap acoustic startle response amplitude”.

### 2.4. Noise Exposure

After deep anesthetizing with sodium pentobarbital i.p. injected, rats wearing foam earplugs (OHRFRIEDEN, Wehrheim, Germany) unilaterally on the right ear were exposed to loud noise at 126 dB SPL with a frequency at 12 kHz for 2 h. This procedure has been demonstrated to enable animals to maintain normal hearing at least in one ear after noise exposure, which is necessary for gap detection [27]. A TW67 speaker (Pyramid Car Audio, Brooklyn, NY, USA) acting as the sound source was placed 10 cm away from the left ear of the rats. A TDT processor and RA 300 amplifier (Alesis, Cumberland RI, USA) was arranged to generate and amplify the tone, respectively. A sound level meter with a condenser microphone was utilized to calibrate the SPL.

### 2.5. RNA-seq

The procedure of the RNA-seq was in appliance with previous literature [28]. The anatomical criteria to identify the auditory cortex of the rat was in accordance with the atlas of Paxinos and Watson. In brief, regions (2.7–5.8 mm posterior to bregma; 6.8–8.67 mm lateral to the midline; 3–4.6 mm ventral to the dorsal surface of the skull) were selected as the target area [29]. Brain samples of the auditory cortex containing both the primary and secondary auditory cortices in the tinnitus and non-tinnitus group (n = 3) were immediately dissected and washed followed by storage in TRIzol reagent (Thermo Fisher Scientific). NanoDrop 2000 (Thermo Fisher Scientific, Wilmington, NC, USA) was used to measure RNA concentration and purity. Assessment of RNA integrity was performed by the RNA Nano 6000 Assay Kit of the Agilent Bioanalyzer 2100 system (Agilent Technologies, Santa Clara, CA, USA). Sequencing libraries were generated with the NEBNext UltraTM RNA Library Prep Kit for Illumina (NEB, Ipswich, MA, USA) according to the manufacturer’s recommendations and index codes were added to assign sequences per sample. Through in-house Perl scripts, raw data (raw reads) in fastq format were processed. In this step, clean data (clean reads) were obtained by removing reads containing ploy-N and a sequence adapter, as well as low-quality reads from raw data. At the same time, Q20, Q30, GC-content, and sequence duplication levels of the clean data were calculated. The clean data were subjected to downstream analyses.

### 2.6. Bioinformatics Analysis

Differential expression analysis of tissue samples was performed with the edgeR. The *p*-value ≤ 0.05 and Fold Change ≥ 1.5 were set as the threshold to detect significant differential expression. Gene Ontology (GO) and Kyoto Encyclopedia of Genes and Genomes (KEGG) enrichment analysis were performed with the GOseq R packages and KOBAS software, respectively. The PPI analysis was performed by submitting differentially expressed genes (DEGs) to the STRING database (http://stringdb.org/ (accessed on 14 August 2022)). Hub genes were visualized and selected by Cytoscape software [30]. To establish the network of protein–protein interaction, the STRING database was used [31,32]. Medium confidence (0.400) of the interaction score was selected as the criterion to identify each interaction [33].

### 2.7. Quantitative PCR

RNA extraction was conducted according to the procedures described previously [30]. After the extraction with RNA extraction reagent (Servicebio, Wuhan, China), the total RNA was used to synthesize complementary DNA using the Servicebio^®^RT First Strand cDNA Synthesis Kit (Servicebio, Wuhan, China). Quantitative PCR was performed using 2×SYBR Green qPCR Master Mix (Servicebio, Wuhan, China). β-actin mRNA levels were used to normalize the mRNA levels of genes selected and the 2^−ΔΔCT^ method was utilized to calculate the fold-change in expression. Table 1 contains a list of all oligonucleotide primers (Servicebio, Wuhan, China) in the experiment.

### 2.8. Statistics

GraphPad Prism software (version 9.0.1, San Diego, CA, USA) was used to analyze the behavioral data. All data are presented as means ± SEM. Statistical significance was confirmed as *p* < 0.05.

## 3. Results

### 3.1. Establishment and Validation of Noise-Induced Tinnitus Rat Model

**Figure 1:**
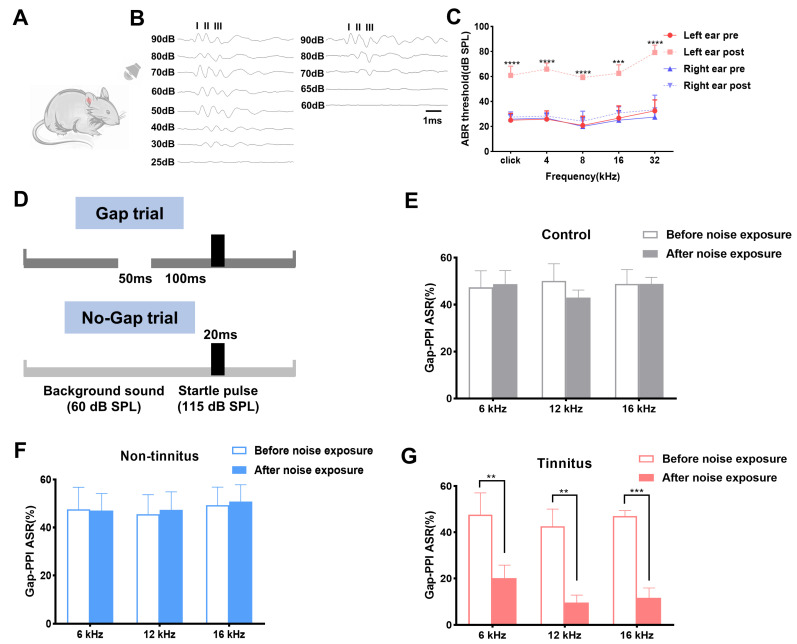
Establishment of noise-induced tinnitus rat model. (**A**) Schematic figure of the experimental setting. (**B**) Typical image of click-ABR threshold of the left ear before (**left**) and after the noise exposure (**right**). (**C**) Click-ABR and pure tone-ABR threshold of both ears before and after the noise exposure. (**D**) The experimental paradigm set in the current work detecting tinnitus. (**E**,**F**) Graphs of Gap-PPI before and after noise exposure for background sounds at 6, 12, 16 kHz in the control and non-tinnitus group (n = 6). (**G**) Graphs of Gap-PPI before and after the noise exposure for background sounds at 6, 12, 16 kHz in the tinnitus group. n = 6. ** *p* < 0.01, *** *p* < 0.001, **** *p* < 0.0001, bar = 1 ms. ABR: auditory brainstem response; PPI: pre-pulse inhibition.

To acquire the tinnitus model, rats wearing earplugs on the right side were exposed to loud noise as demonstrated in Figure 1A. The acoustic trauma by noise exposure was further identified by measuring the threshold of ABRs to a click and tone stimuli on both sides. Figure 1B depicts the significant threshold change of the left ear before and after noise modulation. The hearing threshold of the left ear after noise exposure significantly increased in comparison to that of the pre-noise at a click (pre vs. post, 25.00 ± 2.24 vs. 60.83 ± 3.00, *p* < 0.0001), 4 kHz (pre vs. post, 25.83 ± 2.71 vs. 65.83 ± 2.00, *p* < 0.0001), 8 kHz (pre vs. post, 20.83 ± 3.00 vs. 59.17 ± 0.83, *p* < 0.0001), 16 kHz (pre vs. post, 26.67 ± 4.01 vs. 62.50 ± 2.81, *p* < 0.001), and 32 kHz (pre vs. post, 32.50 ± 3.59 vs. 79.17 ± 2.39, *p* < 0.0001), respectively (Figure 1C). The hearing of the right side was successfully protected in click (pre vs. post, 25.83 ± 1.54 vs. 27.50 ± 1.71, *p* = 0.53), 4 kHz (pre vs. post, 26.67 ± 1.67 vs. 28.33 ± 1.05, *p* = 0.47), 8 kHz (pre vs. post, 20.00 ± 2.89 vs. 24.17 ± 3.27, *p* = 0.34), 16 kHz (pre vs. post, 25.00 ± 3.16 vs. 30.83 ± 2.00, *p* = 0.16), and 32 kHz (pre vs. post, 27.50 ± 2.14 vs. 33.33 ± 4.77, *p* = 0.16), with detection of the gap set artificially in the subsequent continuous background sound. The experimental protocol used to evaluate the animals’ ability to detect the gap is shown in Figure 1D. The Gap-PPI percentage of the control and the non-tinnitus group both displayed no significant change after noise exposure at 6, 12, and 16 kHz frequencies, which is shown in Figure 1D,F. The tinnitus group exhibited a significant Gap-PPI decrease at 6 kHz, which was 20.18 ± 5.61%, in comparison with the control group (47.68 ± 9.38%, *p* < 0.01, Figure 1G). The inhibitory effect of the gap on the acoustic startle (12 kHz) was equally attenuated in the tinnitus group (pre vs. post, 42.65 ± 7.42 vs. 9.61 ± 3.22, *p* < 0.01, Figure 1G). The same tendency was observed in the 16 kHz background sound in which Gap-PPI of the tinnitus group decreased from 47.01 ± 2.43% to 11.66 ± 4.22% (*p* < 0.001).

### 3.2. The Identification of Genes Expressed Differentially between the Tinnitus and the Non-Tinnitus Group

**Figure 2:**
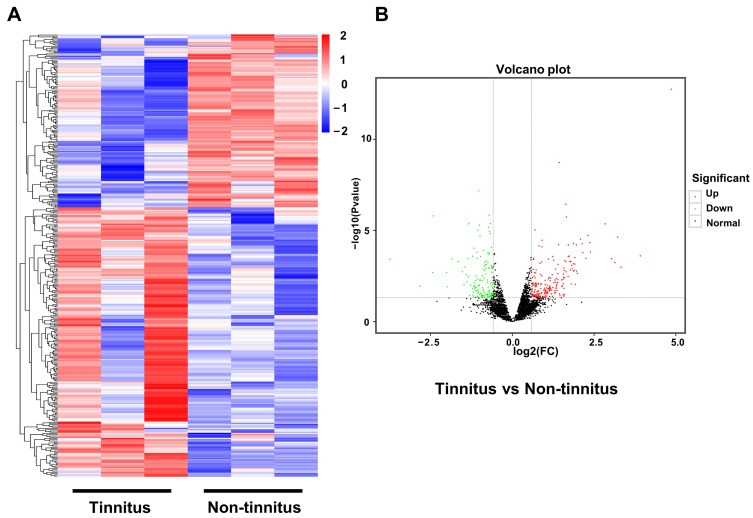
Confirmation of genes differentially expressed in the transcriptional level between the tinnitus and the non-tinnitus group. (**A**) Heatmap of DEGs in the tinnitus (**left**) and the non-tinnitus group (**right**). Blue and red color indicate the down-regulated and up-regulated genes, respectively. (**B**) Volcano image of DEGs between the tinnitus and the non-tinnitus group. The up-regulated genes are displayed in red dots, while the down-regulated genes are shown in green. n = 3. DEGs: differentially expressed genes.

To elucidate the concrete transcriptional profile changes in a noise-induced tinnitus rat model, we performed the RNA-seq of brain samples from the tinnitus and the non-tinnitus group. Since numerous studies regarding the mechanism of tinnitus have found that the auditory cortex played an essential role in its genesis, the auditory cortex was selected as the target area in our research. When a gene exhibited a change greater than 1.5-fold between the tinnitus and the non-tinnitus group, it was included as a DEG. Based on the above criteria, 162 up-regulated genes and 97 down-regulated genes were identified. In Figure 2, the DEGs are displayed in the heatmap (Figure 2A) and the volcano plot (Figure 2B) respectively.

### 3.3. Differentially Expressed Genes Enrichment Analysis

**Figure 3:**
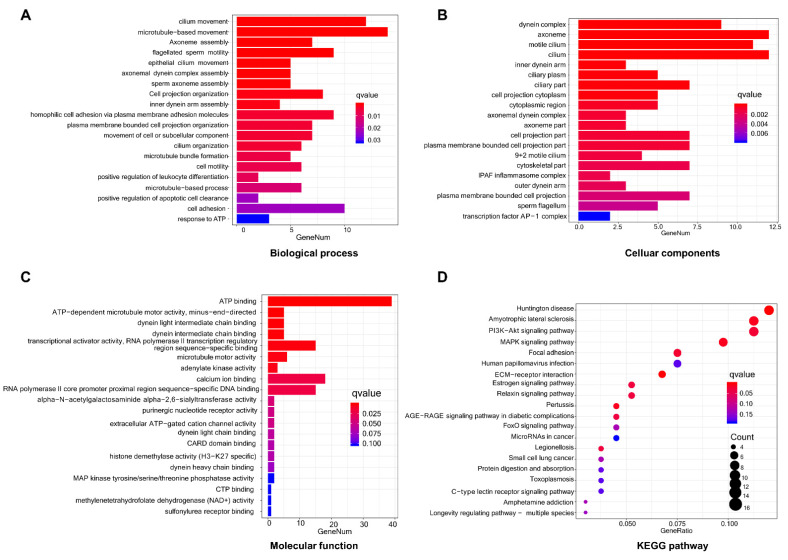
GO and KEGG enrichment analysis of DEGs. (**A**–**C**) Representative columns of GO analysis belonging to biological process (**A**), cellular components (**B**), and molecular function (**C**) category. (**D**) The top 20 enriched pathways of DEGs via KEGG enrichment analysis. GO: Gene Ontology; KEGG: Kyoto Encyclopedia of Genes and Genomes.

The relevant biological functions of DEGs between the tinnitus and the non-tinnitus group were detected by GO enrichment analysis. Enrichment terms that displayed significance in the category of biological process, cellular components, and molecular function are depicted in Figure 3A–C. Cilium movement, microtubule-based movement, axoneme assembly, flagellated sperm motility, and epithelial cilium movement made up the top five terms in the BP category. The dynein complex was the most enriched cellular components process, followed by axoneme, motile cilium, cilium, and inner dynein arm. For the molecular function process, the top five enriched terms were ATP binding, ATP-dependent microtubule motor activity (minus-end-directed), dynein light intermediate chain binding, dynein intermediate chain binding, and transcriptional activity (RNA polymerase II transcription regulatory region sequence-specific binding).

To further elucidate the underlying pathways involved in the DEGs between the tinnitus and the non-tinnitus group, we performed KEGG analysis (Figure 3D). The potential pathway identified includes: Huntington’s disease, Amyotrophic lateral sclerosis, PI3K-Akt signaling pathway, MAPK signaling pathway, Focal adhesion, ECM-receptor interaction, Estrogen signaling pathway, Relaxin signaling pathway, Pertussis, and AGERAGE signaling pathway in diabetic complications.

### 3.4. Construction of Protein–Protein Interactions Network and the Selection of Hub Genes

**Figure 4:**
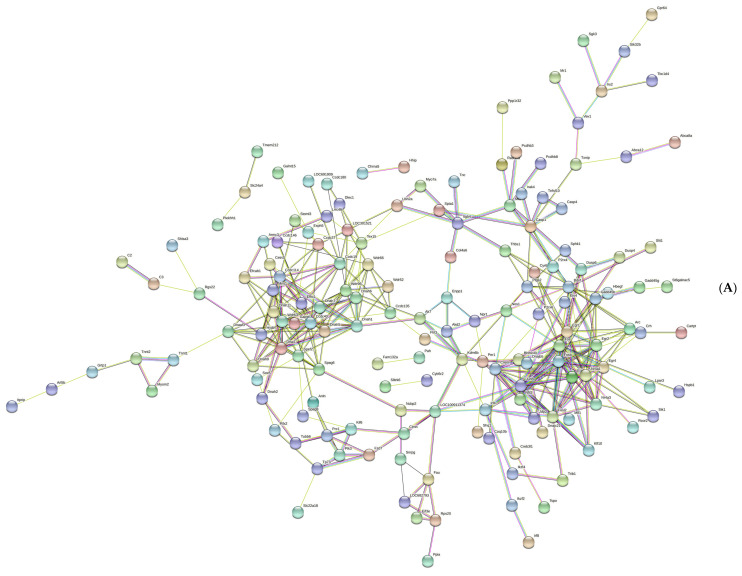

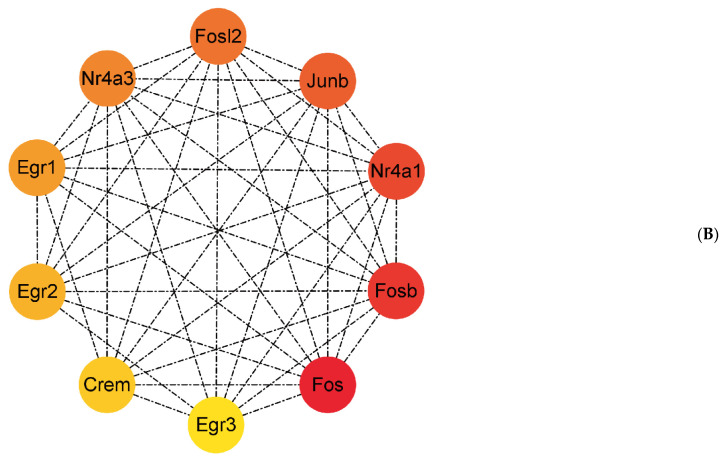
The network of protein–protein interaction. (**A**) The protein–protein interaction network was established by the STRING database. (**B**) The hub genes were selected in this study. The red and yellow color mean the highest to lowest degree among the ten hub genes.

Figure 4A shows a total of 229 nodes and 373 edges in the PPI network, with an average node degree of 3.26. The cytoHubba plug-in was used to calculate the hub genes. Ten genes were identified, with net degree ranking demonstrated in Figure 4B, namely, *Fos, Fosb, Nr4a1, Junb, Fosl2, Nr4a3, Egr1, Egr2, Crem,* and *Egr3.*

### 3.5. Verification of the Hub Genes

**Figure 5:**
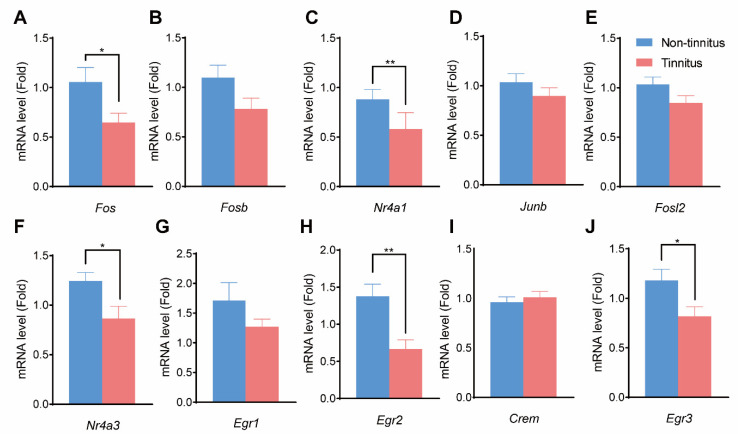
qPCR results of the selected hub genes. (**A**–**G**) show the mRNA level changes between the non-tinnitus and the tinnitus group including *Fos* (**A**), *Fosb* (**B**), *Nr4a1* (**C**), *Junb* (**D**), *Fosl2* (**E**), *Nr4a3* (**F**), *Egr1* (**G**), *Egr2* (**H**), *Crem* (**I**), and *Egr3* (**J**). n = 5, * *p* < 0.05, ** *p* < 0.01. qPCR: quantitative PCR.

qPCR was performed to detect the expression of the selected genes mentioned above. The expressions of *Fos* (*p* < 0.05, Figure 5A), *Nr4a1* (*p* < 0.01, Figure 5C), *Nr4a3* (*p* < 0.05, Figure 5F), *Egr2* (*p* < 0.01, Figure 5H), and *Egr3* (*p* < 0.05, Figure 5J) were significantly decreased in the tinnitus group compared to the non-tinnitus group. On the other hand, *Fosb* (*p* = 0.09, Figure 5B), *Junb* (*p* = 0.29, Figure 5D), *Fosl2* (*p* = 0.12, Figure 5E), *Egr1* (*p* = 0.21, Figure 5G), and *Crem* (*p* = 0.56, Figure 5I) showed no statistical difference between the two groups.

## 4. Discussion

Tinnitus is an intractable disease that disrupts patients’ lives and is a burden to society, especially when it progresses from acute to chronic status (>6 months) [34]. Due to its elusive mechanism and confusing pathogenesis, effective treatments against tinnitus are still being developed. In the current study, we first established a rat tinnitus model by exposing animals to loud noise and subsequently confirmed the generation of noise-induced tinnitus using gap-induced PPI of the acoustic startle response method. Then the RNA-seq was utilized to analyze the genetic change between the tinnitus and the non-tinnitus group. A total of 259 genes related to tinnitus comprising 162 up-regulated and 97 down-regulated genes was found. GO and KEGG analysis were performed on the DEGs to shed light on their potential function and signaling pathways. We discovered five hub genes by interpreting the PPI network of genes expressed differentially. These genes may serve as potential treatment targets and were verified at the transcriptional level.

Previous research showed that tinnitus frequency was similar to the noise frequency applied in the experimental paradigm [26]. In the present study, animals showed tinnitus-related behavior at 6, 12, and 16 kHz, which was adjacent to the frequency of loud noise at 12 kHz. Among the top up-regulated genes, which included Spata18, Dnah6, AABR07015078.2, AC128059.4, AC128059.4, AABR07015055.1, Stk32b, Lrrc74b, Slc22a18, and Stoml3, Spata18 was a major participant in the cellular response to DNA damage stimulus. Spata18 can act as a potential target for tinnitus treatment because it is involved in the autophagy process, which undergoes the anti-tinnitus effect of melatonin [35]. Meanwhile, down-regulated genes between the tinnitus and the control group were Car3, Egr2, Bcl6b, C1qtnf12, Cartpt, LOC108348062, Nr4a3, Gprc5a, LOC103690128, and Angptl6. Carbonic anhydrases are considered essential for neural transmission by activation and modulation of protons and bicarbonate ions, which may alleviate neurodegenerative disorders [36,37]. Down-regulation of the Car3 gene suggests that attenuated transmission of neural signals such as glutamate and GABA in the auditory cortex may mediate the generation of tinnitus [38,39].

One of the most enriched pathways in tinnitus induced by loud noise is the PI3K-AKT signal pathway. This plays an important role in regulating multiple biological processes in the central nervous system, including synaptic plasticity, neuron survival, cellular proliferation and differentiation, neurogenesis, and autophagy [40]. The activated PI3K-AKT pathway can be involved in facilitating neuron survival and growth through apoptosis inhibition [41]. Meanwhile, oxidative stress can be modulated by the PI3K-AKT pathway via up- or down-regulating related molecular processes including FoxO3a, mTOR, and GSK-3 [42]. All the processes mentioned above are common in neurodegenerative diseases that usually exhibit the symptom of tinnitus [43]. KEGG pathways involved in Amyotrophic lateral sclerosis and Huntington’s disease are mainly enriched, which further verified that tinnitus is a subtype of neurodegenerative disorders and may share similar characteristics and mechanisms [44,45].

Investigating the hub genes encoding the protein interactions may be helpful to shed light on the mechanism mediating the generation of noise-induced tinnitus. Out of the 10 identified genes, the *Fos* gene ranked first and demonstrated the highest degree. Our results showed a decreased expression of the *Fos* gene in the tinnitus group after noise exposure. This is different from Qin’s work, which observed increased c-fos expression in tinnitus animals [46]. One of the contributory factors to this phenomenon is that the types of tinnitus in our study and Qin’s study are different. Drug-induced tinnitus by salicylate usually takes effect within several minutes and diminishes after about 72 h, which overlaps with the time frame of c-fos expression. Results from research investigating tinnitus drugs may observe the up-regulation of *Fos* since the *Fos* gene is rapidly expressed in the early stage (0.5 to 4 h later than the stimulus) [46,47]. Another explanation for this novel finding is that the decreased *Fos* gene expression in the auditory cortex may have an integral excitatory effect. Abundant interneurons including excitatory cells (glutamatergic) and inhibitory cells (GABAergic) located in this area constitute the balanced network of excitation and inhibition. If the down-regulated *Fos* genes in the inhibitory interneurons make a greater contribution to the integral output than those in the pyramidal neurons, the comprehensive effect of the auditory cortex will be excitatory, which is in agreement with the findings in other studies [48]. Another gene to be considered is *Nr4a1* which belongs to the category of immediate-early genes. The gene is also associated with neural activity, influences neural pathway maturation, and modulates cell function [49,50]. As a subtype of orphan nuclear receptors, it can prevent the development of inflammation and generate anti-inflammatory effects [51,52]. Down-regulation of the *Nr4a1* gene means that noise-induced tinnitus may derive from neural inflammation in the central nervous system [53].

There are still several potential limitations of our study that need to be mentioned. Discovering genes that encode the occurrence and persistence of tinnitus will serve as a guide for physicians to detect tinnitus susceptibility in people and subsequently enable researchers to explore advanced interventions [54]. However, this study mainly focused on the occurrence but not the persistence of tinnitus, especially when it progresses into the chronic phenotype, which lasts longer than 6 months [55]. Although we utilized loud noise to generate a tinnitus model that lasts longer than drug-induced tinnitus to simulate the disease occurring in patients, this falls far short of the clinical practice because there is a lack of an explicit distinction between noise- and drug-induced tinnitus in the real medical environment. Moreover, the hub genes in our study were acquired from rodents instead of human specimens, so caution should be paid when extrapolating these results from bench to bedside because of different genetic backgrounds [56]. In addition, genes related to tinnitus in our research were only verified at the transcriptional level. The possibility that they translate into functional proteins to regulate tinnitus needs to be further investigated.

## 5. Conclusions

In conclusion, the current research found a total of 259 genes (162 up-regulated and 97 down-regulated) related to tinnitus. Most DEGs were enriched in the neurodegenerative-disease-related pathway, making it possible to treat tinnitus using neurodegenerative diseases for reference. In addition, since the Fos gene ranked first with the highest degree after noise exposure, it may be a potential target for the modulation of noise-induced tinnitus. Further validation should be conducted to verify its effects on tinnitus so as to provide novel treatments to alleviate noise-induced tinnitus in patients.

## Figures and Tables

**Table 1:** Primer sequence of genes selected for qPCR analysis.

Genes	Forward	Reverse
*Fos*	TGCTATGTTGCCCTAGACTTCG	GTTGGCATAGAGGTCTTTACGG
*Fosb*	AGAAACCGTCGGAGGGAGCT	CCGTCTTCCTTAGCGGATGTT
*Nr4a1*	TTGGAAAGGAAGATGCCGG	TGTCTATCCAGTCACCAAAGCC
*Junb*	ACGACTACAAACTCCTGAAACCC	TGATCCCTGACCCGAAAAGTAG
*Egr1*	CCAAACTGGAGGAGATGATGCT	GACTCTGTGGTCAGGTGCTCGTA
*Fosl2*	CTCAGCAGGGATGGACAAGAC	GGTGAAGACAAGGTTTGAAGTGC
*Nr4a3*	GACTTTCTATCAGGTCAAACACTGC	AGGCAGGCTAAGGCTTGGATA
*Egr2*	GCCGTAGACAAAATCCCAGTAAC	AAGATGCCCGCACTCACAATA
*Crem*	TGAGGACAAATGTAAGGCAAATGAC	ACCGATGGATGTGGTGTCTGAAT
*Egr3*	AGATGGCTACAGAGAATGTGATGG	CTAATGATGTTGTCCTGGCACC
*β-actin*	TGCTATGTTGCCCTAGACTTCG	GTTGGCATAGAGGTCTTTACGG

## Data Availability

Not applicable.
